# Relationship between Capillary Wettability, Mass, and Momentum Transfer in Nanoconfined Water: The Case of Water in Nanoslits of Graphite and Hexagonal Boron Nitride

**DOI:** 10.1021/acsami.4c10738

**Published:** 2024-10-08

**Authors:** Lois Smith, Zixuan Wei, Christopher D. Williams, Mara Chiricotto, Claudio Pereira da Fonte, Paola Carbone

**Affiliations:** † Department of Chemistry, University of Manchester, Oxford Road, M13 9PL Manchester, U.K.; ‡ Department of Chemistry, University of Liverpool, Crown Street, L69 7ZD Liverpool, U.K.; § Division of Pharmacy and Optometry, School of Health Sciences, University of Manchester, Oxford Road, M13 9PL Manchester, U.K.; ∥ The Hartree Centre, STFC Daresbury Laboratory, WA4 4AD Warrington, U.K.; ⊥ Department of Chemical Engineering, University of Manchester, Oxford Road, M13 9PL Manchester, U.K.

**Keywords:** nanoconfined water, graphite, hBN, diffusion, flow rate, Hagen–Poiseuille theory

## Abstract

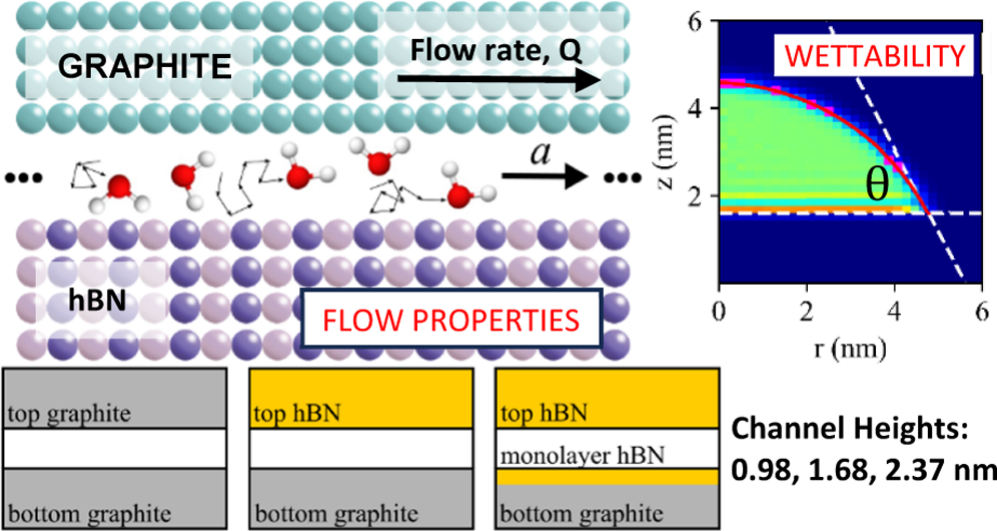

The flow of water confined in nanosize capillaries is subject of intense research due to its relevance in the fabrication of nanofluidic devices and in the development of theories for fluid transport in porous media. Here, using molecular dynamics simulations carried out on 2D capillaries made up of graphite, hexagonal boron nitride (hBN) and a mix of the two, and of sizes from subnanometer to few nanometers, we investigate the relationship between the wettability of the wall capillary, the water diffusion, and its flow rate. We find that the water diffusion is decoupled from its flow properties as the former is not affected either by the height or chemistry of the capillary (except for the subnanometer slits), while the latter is dependent on both. The capillaries containing hBN show a reduced flow rate compared to those that are purely graphitic, likely due to the high friction coefficient between water and hBN. Such resistance to the flow is, however, at its maximum in the smallest capillary and lower for larger ones. Finally, we show that the flow rate values obtained from the Hagen–Poiseuille theory are almost always smaller than those obtained from simulations, indicating that either the slip length or the viscosity of nanoconfined water could be substantially different from the bulk values.

## Introduction

The behavior of water at the nanoscale has recently garnered great interest for engineering applications such as water desalination, purification, and energy storage and conversion. This is due to the enhanced interfacial effects typical of nanofluidic devices, where most of the fluid molecules interact with the surface of the nanocapillaries. Therefore, constructing these devices from materials that aid in fast and efficient water transport is of the upmost importance. The ability to manipulate and manufacture nanocapillaries has greatly improved in the last 10 years and this has tremendously helped in identifying not only materials but also channels’ geometries and sizes that facilitate water transport.^1−4^ In particular, specific allotropes of carbon (carbon nanotubes, graphite/graphene) and the hexagonal crystalline form of boron nitride (hBN) have proved to be potentially disruptive materials in the field.^5−14^

The exact mechanism by which water and electrolyte flow within nanocapillaries is still debated since the fluid transport data are scattered and difficult to rationalize.^5,15−19^ Experiments, theory, and simulations have pointed out that several different aspects of the device and liquid properties affect transport data. These include interfacial properties such as friction coefficient and slip length but also liquid properties such as density and viscosity which differ from the bulk values and depend on the level of confinement.^4,20−27^ What is particularly intriguing is that the interfacial tension, despite changing under confinement^28^ and being correlated to the values of the slip length,^29,30^ does not affect the flow data. This counterintuitive phenomenon has been observed in experiments involving graphene and hBN with both nanotubes and nanoslits and has been attributed to the difference in water/surface friction coefficients, λ, between the two materials.^4,31−33^ Indeed, despite their equivalent crystal structure (hexagonal), graphene and hBN have significantly different surface charge distributions due to the difference in the atomic partial charge between the boron (B) and the nitrogen (N) in hBN compared to the homogeneous (and very small) surface charge of graphene. Simulations have indicated that this difference is enough to lead to an increase in the friction coefficient (and thus slip length) of water/hBN compared to water/graphene between three to five times.^33,34^ Although simulations agree on the fact that the water/hBN interface is characterized by a higher friction coefficient than that of water/graphene, they do not fully concur on the reasons behind this difference and its magnitude. Ab initio molecular dynamics (MD) simulations^33^ mainly pinpointed the increase to the more corrugated surface energy landscape of boron nitride compared to graphene and find that the friction coefficient of the former is three times larger than the latter. MD simulations^34^ highlighted instead the role of the solid/liquid electrostatic interactions (almost absent in the graphene but substantial for hBN) in increasing the value of λ yet not appreciably affecting the wetting behavior. This latter observation not only clarifies why, despite the wetting properties of the two surfaces being similar, the corresponding λ is so different but also indicates that this counterintuitive result might be relevant only for polar liquids, such as water, but less so for apolar liquids, with important consequences, for example, on separation processes.

Despite this work, the physics driving the value of the friction coefficients between water and graphitic surfaces and its effect of fluid transport properties is all but clear.^17^ Recently, a new contribution to friction arising from the coupling of charge fluctuations in the liquid to electronic excitations in the solid has also been identified.^35^ The new quantum theory of the solid–liquid interface that incorporates this effect could help to explain interesting phenomena such as the dependence of γ on the surface curvature and on the number of graphene layers. The role of the electronic structure of the surface on the friction forces, and the importance to untangle it from classical effects,^36^ has also been confirmed by the fact that introducing atomic-scale structural and chemical defects in the hBN surface is enough to increase its friction coefficient by almost an order of magnitude.^37^ Experiments have also clearly indicated that nanochannels made of graphite and hBN have different flow data, although with different conclusions. Experiments carried out on hBN nanotubes, for which more data than for 2D nanoslits are available, provide contradictory results, some indicating enhanced and others indicating reduced water transport compared to CNTs.^5,16^ Recent experiments conducted on 2D slits of graphite, hBN, and a mix of the two instead hint, at least for this capillary geometry, to a reduced flow when hBN sheets are included in the fabrication of the conduit.^32^ Many factors can affect the spread of experimental data including structural and chemical defects of the surface as pointed out above (which are often the results of the method followed to synthesize the material), the size and geometry of the capillaries but also the chemical nature of the substrates, and the type of measurements used to obtain the flow data.

In this paper, we perform MD simulations of water flowing inside nanocapillaries formed by both graphite, hBN, and a mix of the two in a molecular model that reproduces the experimental setup reported by Keerthi et al.^32^ Due to the wide range of the experimental data, we do not expect to achieve quantitative agreements with the experimental data, but our simulations can provide a systematic exploration of how surface wetting, confinement, and chemistry of the slit affect the water structural and transport properties. By comparing the capillary wall wettability, the water diffusion coefficient, and its volumetric flow rate, we clarify that the wall wettability does not correlate with the water flow which is instead dominated by a confinement- and chemistry-dependent solid/liquid friction coefficient. Finally, we find that at such small confinement, the mass and momentum transfer are decoupled since while the water diffusion coefficient is almost unaffected by the capillary chemistry and height (the latter for capillary larger than 0.94 nm), the volumetric flow rates are.

## Methods

### Capillary Construction and Model Details

**Figure 1:**
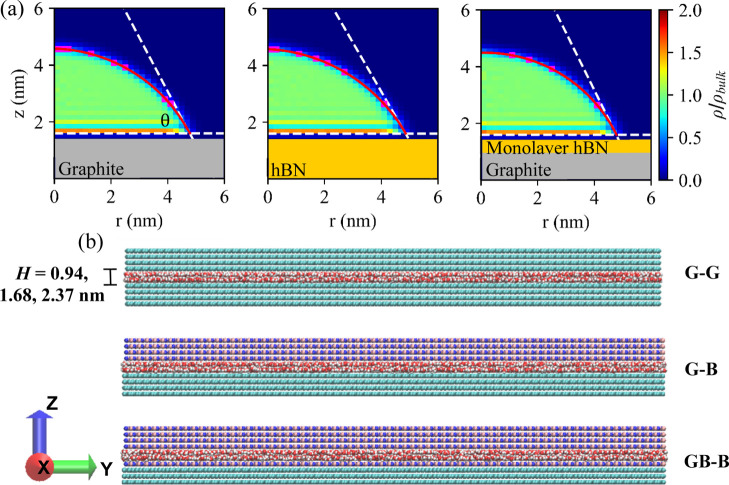
(a) Droplet simulations and corresponding water contact angle (WCA) measurements on graphite (left), multilayer hBN (center), and hBN/graphite hybrid (right). These surfaces were chosen to correspond to the three types of chemistry present in the channels. A circular fit to the droplet liquid–vapor interface is displayed in red and the white dashed lines represent the placement of the water contact layer and corresponding tangent to the circular fit used to measure the WCA. (b) Schematics of the three channel chemistries: graphite–graphite, G–G (top); graphite–hBN, G–B (middle); and graphite/hBN monolayer–hBN, GB–B (bottom). Equilibrated water is depicted in each channel.

The 2D channels simulated in this work are made of a combination of eight approximately 10 × 30 nm sheets of graphene or monolayer hBN with channel height, *H*. Each system is periodic in all directions and the channel walls are frozen throughout all simulations. The chemistry of each channel is displayed in Figure 1. The choice of channel height *H*, defined by the distance between the centers of the atoms composing the top and bottom slit walls, was dictated by experimental^38^ and our own simulation data^39^ which indicated that these channels are mechanically stable when *N* = 1, 2, and 3 graphene flakes are removed from a slab of flexible graphite, respectively, and the resulting capillary is filled with water. However, since the mechanical stability of the water filled channels have been tested only for those of pure graphite, further MD simulations have been carried out for capillaries containing hBN sheets following the same procedure as described in Williams et al.^39^ The details of these simulations are reported in the Channel Height Validation section of the Supporting Information and the results indicate that the heights of water filled capillaries do not depend on the number of hBN sheets included but only on the number of graphene sheets removed to create the channel. Thus, the mechanically stable channel heights were the same as those previously determined for a purely graphitic channel.

The procedure for creating and filling the channels of predefined height is taken from Dix et al.^40^ and summarized here. First, the channels were constructed with flat, defect-free sheets of graphene and/or hBN with the configurations and details specified in Figure 1 and Table 1, respectively. The total number of sheets that form the top and bottom of the channels was always *n* = 4 with an interlayer spacing of 0.335 nm. Two bulk water reservoirs were placed in contact with the channels and the system was simulated in the *NVT* ensemble (constant number of particles, volume, and temperature) until the 2D water density inside the channels, ρ_2D_, reached a constant value. We found that approximately 20 ns was sufficient time for this condition to be met which is consistent with the previous work.^28^ The 2D water density values reported in Table 2 agree with previous simulations ran with flexible graphitic channels,^39^ indicating that the simplification of keeping the channel wall frozen is acceptable. The reservoirs were then removed, and the box size was accordingly reduced such that an infinitely long channel was formed. Each simulation was performed with the GROMACS software package^41^ at a temperature of 298.15 K, held constant by the Nosé–Hoover thermostat with a time constant of 0.5 ps. The equation of motion was integrated with the Verlet algorithm with a time step of 2 fs. The nonbonded interactions were handled by the 12–6 Lennard-Jones (LJ) potential which is smoothly switched to zero between 1 and 1.2 nm, and the long-range electrostatic interactions were handled with the particle mesh Ewald with a Fourier grid spacing of 0.16 nm. During the MD simulations, the graphene carbon, hBN boron, and nitrogen atoms have nonbonded interactions with water atoms modeled by the 12–6 LJ potential. The LJ parameters for the carbon atoms are taken from Cornell et al.^42^ except for the C-OW cross-interaction parameter of epsilon = 0.4785 kJ/mol which is taken from Werder et al.^43^ As we will show later, this combination of force field parameters produces a water/graphite contact angle within the experimental range.

**Table 1:** Simulation Box Details

channel type	number of hBN sheets	number of graphene sheets	*x*–*y* box dimension (nm)	*H* (nm)
G–B	4	4	10.07 × 30.204	0.94
				1.68
				2.37
GB–B	5	3	10.07 × 30.204	0.94
				1.68
				2.37
G–G	0	8	10.07 × 30.204	0.94
				1.68
				2.37

**Table 2:** 2D Density (ρ_2D_) and WCA for the Various Substrate Types and Capillary Heights (*H*)^a^

*H* [nm]	capillary type	ρ_2D_ [mol/nm^2^]	substrate type	WCA [deg]
0.94	G–G	20.51		61.29 ± 0.73
1.68		43.81	G	
2.37		66.58		
0.94	G–B	23.96		60.34 ± 2.23
1.68		44.62	GB	
2.37		66.77		
0.94	GB–B	24.63		63.17 ± 1.76
1.68		45.41	B	
2.37		67.61		

a The error on the WCA values was calculated by block averages.

The parameters for the boron and nitrogen atoms in the hBN sheet are taken from Wagemann et al.^44^ Partial charges of +0.3*e* and −0.3*e* were applied to the boron and nitrogen atoms, respectively. Note that the BN bond length was slightly modified from a value of 0.1446 nm, reported in the original forcefield, to a value of 0.1454 nm. This was necessary to avoid an atom mismatch at the box *y*-boundaries (longest channel dimension) when both hBN and graphene sheets were used in building the channel, as we anticipated that an atom mismatch in this dimension would impact the results to a noticeable degree. This resulted in a stretched C–C bond of approximately 0.252 nm at the *x*-boundary of each graphite/hBN mixed channel. The new bond length was used in all simulations that include hBN, and we found that this did not impact the wettability of the hBN surface compared to a previous work using the original bond length.^44^ The water molecules were modeled by the TIP4P/2005 model^45^ which is a rigid, 4-site nonpolarizable model that can accurately reproduce the liquid/vapor surface tension^46^ and has been used previously to study water under confinement.^28,39,40,47,48^

### Capillary Simulations and Calculation of the Diffusion Coefficient

To determine the water self-diffusion coefficient in the capillaries, we calculated the oxygen mean square displacement (MSD) over an 8 ns *NVT* simulation at 298.15 K of an equilibrated channel of each chemistry and height. We calculated the in-plane (*x*–*y*) diffusion coefficient, *D*_∥_, according to the Einstein relation
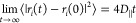
1where *r*_*i*_(*t*) and *r*_*i*_(0) refer to the positions of the *i*-th oxygen atom at time t and 0, respectively. A linear regression was performed on the MSD in the region 100 ps to 1 ns to extract *D*_∥_.

### Droplet Simulations and Contact Angle Calculation

To estimate the wettability of the capillary wall, we calculated the contact angle of a water droplet in contact with a surface of one of the three chemistries: 5-layer graphite, 5-layer hBN, or 4-layer graphite with a monolayer hBN. To create the droplet, we initially placed a cubic box of solvent (5 nm length) composed of 4139 TIP4P/2005 water molecules just above a surface of approximately 24 × 24 nm in the *xy* plane. The total simulation box was 50 nm in the *z*-direction. We then performed an MD simulation in the *NVT* ensemble at 298.15 K for an equilibration period of 2 ns. Periodic boundary conditions were applied in the three directions. The water/surface contact angle was then calculated over a further 2 ns production simulation. The interaction parameters for these systems are the same as those listed in the Capillary Construction and Model Details section. The WCA was measured following the procedure outlined by Zhang and coauthors,^49^ for which we provide a brief overview. First, we obtain an *r*–*z* density profile of the droplet in a cylindrical coordinate system (*r*, ϕ, *z*) with a normal line through its center of mass as a reference axis. This was done by projecting the three-dimensional data onto two-dimensional bins and averaging them over ϕ. The liquid–vapor interface was then identified by selecting the bins that had approximately half the bulk density of the droplet. The WCA was measured as the angle between the solid–liquid contact layer and a tangent fitted to this interface. Plots depicting the WCA measurements are provided in Figure 1a.

### NEMD Simulations and Volumetric Flow Rate Calculations

To extract the flow data, a Poiseuille flow was generated in the nanochannels by applying a constant acceleration (in-plane direction) to each atom of the water molecules. Each capillary was simulated in *NVT* for a total of 4 ns with a range of acceleration between 3 × 10^11^ and 2 × 10^12^ m/s^2^ in line with previous works on water flow through graphite nanopores of various heights^28^ and carbon nanotubes.^50^ To extract the values of water flux across each channel, at each time step, we counted the number of oxygen atoms crossing an imaginary plane perpendicular to the flow and placed at the center of the channel. To calculate the net flux, we subtracted from the count the number of molecules that cross the plane in the opposite direction to that of the applied acceleration. The volumetric flow rate, *Q*, was then obtained multiplying the net flux by the cross-sectional area of each channel. The data was averaged over the last 3 ns of the simulation. In order to test whether the use of the Nosé–Hoover thermostat affects the value of the flow rates,^51,52^ we performed also simulations in the microcanonical ensemble (*NVE*) for three graphene channels. The results (Figure S3 in the Supporting Information) indicate that for these accelerations, the effect is negligible.

## Results and Discussion

### Wetting, Diffusion, and Water Structure

We report the WCAs for 3 different substrate chemistries in Table 2. We note first that the wettability of all surfaces, graphite, hBN, and graphite/hBN hybrid, is very similar, with WCA measurements falling in the range of approximately 60–63° with overlapping error bars. Second, the results agree qualitatively with previous WCA measurements performed using the same hBN forcefield^44^ and also recent experimental works with multilayer hBN and graphite, although in the literature, a wider range of values are reported.^53−55^ The uncertainty on the experimental value can be attributed to a combination of differing methods for the WCA measurements, ambiguity over the identification of the water contact layer, and possible contamination of the surfaces such as the deposition of hydrocarbons. Despite this, our results agree qualitatively with other works citing a similar wettability between graphite and hBN. Table 2 also reports the value of the water density in the channels. To avoid having to define an effective channel height, which for very narrow nanochannels depends strongly on the volume occupied by the atoms at the edge of each wall, the density data are reported as the number of molecules per unit of area . The results indicate that capillaries with the same size but different chemistry have different water density. The trend in ρ_2D_ across channel types can be explained by the stronger interactions (van der Waals and Coulombic) that the water in channels have with hBN sheets compared to those with graphite (only van der Waals). The difference between the water densities decreases as *H* increases as the larger the channel, the less relevant the strength of the water/surface interactions.

**Figure 2:**
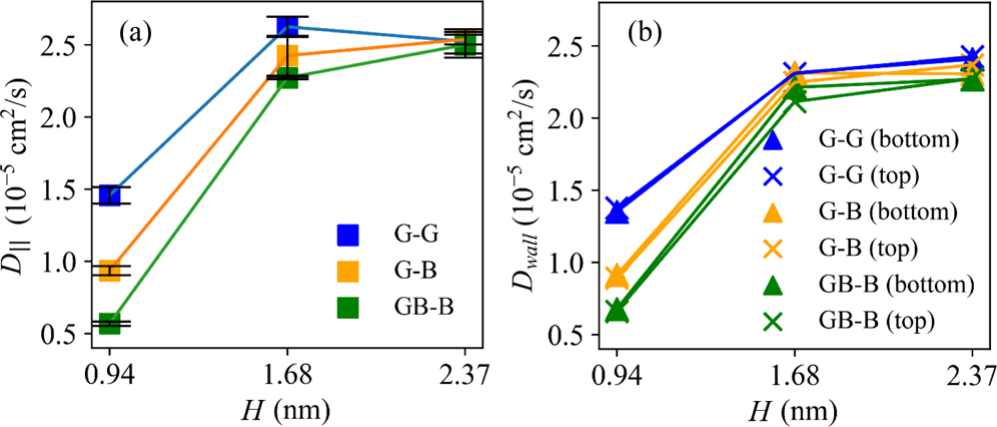
(a) In-plane 2D water self-diffusion coefficient as a function of capillary size (*H*). (b) 2D water self-diffusion coefficient for the water layer closest to the bottom [▲ (blue, orange, and green)] and top [× (blue, orange, and green)] of each channel chemistry. See Figure 1b for the chemistries of the top and bottom channel walls. Error estimates were obtained by calculating the difference in the MSD over the two-halves of the fit region. Error bars were not provided in (b) as their size is of the order of the symbol size.

The 2D diffusion coefficients (calculated for the simulations with no applied acceleration), *D*_∥_, are reported in Figure 2. The values, which for the graphitic channels are comparable to those obtained for graphitic flexible and rigid channels of similar height,^28,39^ show that the diffusion of water is affected by the channel height but also by the channel chemistry mainly for the smallest capillary sizes for which *D*_∥_ is smaller than the bulk value of 2.2 × 10^–5^ cm^2^ s^–1^ reported for TIP4P/2004.^45^ In agreement with our previous work, for graphitic channels larger than *H* = 0.94 nm, *D*_∥_ increases toward the bulk value and reaches 2.4 × 10^–5^ cm^2^ s^–1^ for *H* = 2.37 nm in agreement with our previous data for channels of similar heights.^28^ The capillaries containing hBN show a similar trend, but the inclusion of the hBN layer clearly reduces the diffusion in the small and medium size channels but not in the largest ones where *D*_∥_ converges. The effect is more pronounced for the capillaries containing hBN on both walls.

The reduced diffusion coefficient calculated in the smallest channels can be explained with the fact that in such narrow slits, the water dynamics is affected by the interactions with both capillary walls since *H* is smaller than the LJ and Coulomb cutoff distance. This effect is the largest in the smallest channels containing hBN, where the water molecules interact with the wall atoms also via the electrostatic interactions. To check whether the *D*_∥_ of the only water molecules in direct contact with the capillary wall is affected by the wall chemistry, we calculated their diffusion coefficient separately, *D*_wall_, using
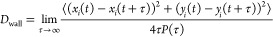
2where *P*(τ) is the survival probability, that is, the probability that a water molecule resides in the water slab in direct contact with the wall during the time interval *t* + τ.^55,56^ The thickness of the slab is chosen according to the minima of the peaks in the oxygen density profiles calculated across each channel (see Figure S4). The values of *D*_wall_, reported in Figure 2b, were calculated for the layers of water in contact with the two capillary walls, separately.

Overall, the trends of *D*_wall_ and *D*_∥_ with *H* across capillary chemistries are the same. Such a trend follows that observed for the water density values reported in Table 2 where we show that for the same *H*, graphitic-type channels have the lowest density and GB–B ones have the highest but also that this difference decreases with increasing channel height. Moreover, the results indicate that the diffusion of this “interfacial” water within capillaries with asymmetric wall chemistry (i.e., G–B and GB–B) is the same irrespective of which wall the water is in contact with (i.e., G or B or GB). These data seem to indicate that the main factor affecting the mass transfer across the channels is the channel water density and, therefore, the degree of confinement rather than the chemistry of the wall. It is also interesting to observe that the density profiles reported in Figure S4 in the Supporting Information show that the layering of the water in the slit is also dependent only on the value of confinement (i.e., H) and not on the channel chemistry. This result, as pointed out by Zangi and Mark,^57^ is because the organization of the water in the channel is a purely excluded volume effect and is not due to any specific interaction with the atoms of the wall.

To examine whether there is a link between the diffusion coefficients and the water structure, we calculated the average water orientation parameter, ⟨*S*⟩, per layer of water in each channel. The water layers were defined based on the corresponding oxygen density profiles (see Figure S4 of the Supporting Information). ⟨*S*⟩ is defined as the expectation value of the second-order Legendre polynomial, *P*_2_, as a function of cos(θ)
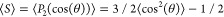
3where θ is the angle between the water dipole moment and a director defined perpendicular to the channel walls. A value of *S* = 1 indicates that the dipole is perfectly aligned with the director, *S* = 0 indicates random alignment, and 0 < *S* < 1 represents partial alignment. A special case arises at *S* = −1/2 which denotes anti-alignment, where the dipole is perpendicular to the director.

**Figure 3:**
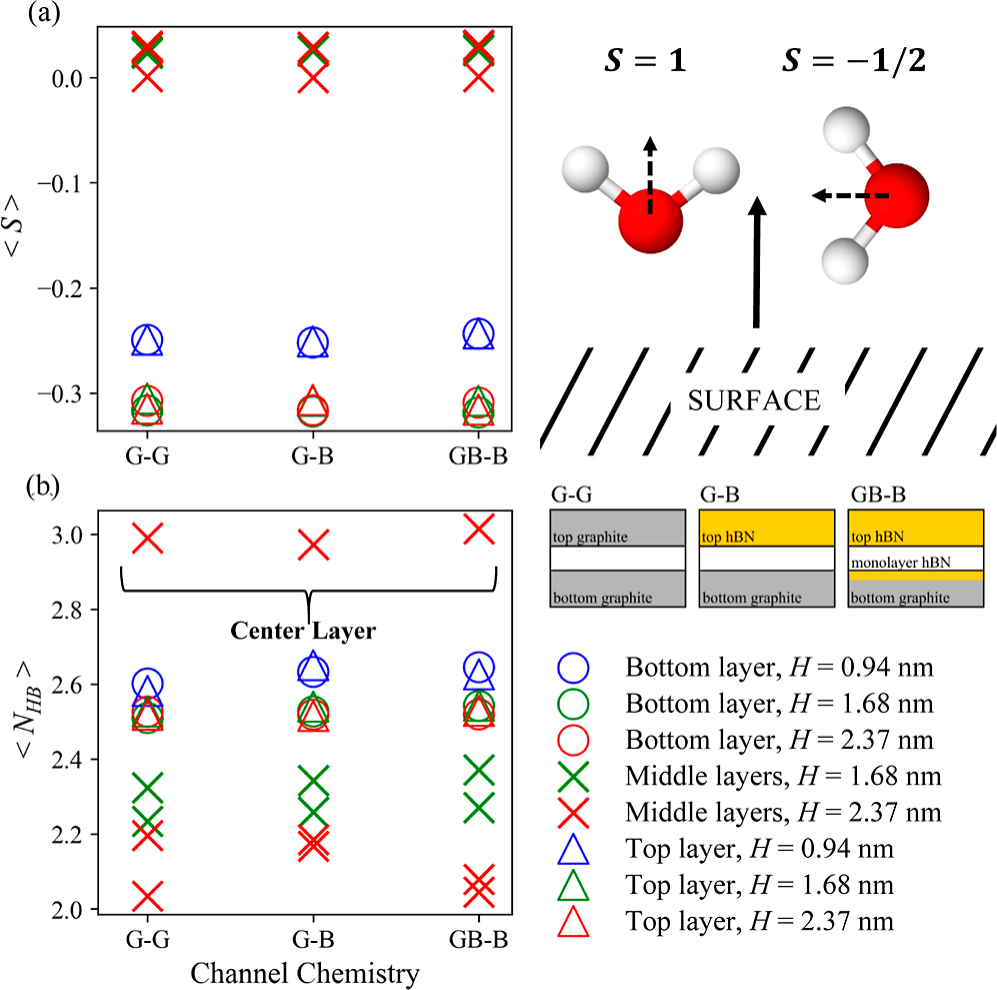
(a) Average order parameter, ⟨*S*⟩, per water layer for each channel and depiction of water molecule orientations displaying alignment (*S* = 1) and anti-alignment (*S* = −1/2) to a director perpendicular to the channel walls (filled black arrow). (b) Average number of hydrogen bonds per water molecule for each channel. Values for both ⟨*S*⟩ and ⟨*N*_HB_⟩ are displayed for heights *H* = 0.94 nm (blue), 1.68 nm (green), and 2.37 nm (red). Results are plotted separately depending on whether the water layer is the closest to the bottom (circles) or top (triangles) channel walls or in the middle (crosses) in the channel. A diagram of the channel chemistries is included to aid the reader.

Figure 3a shows ⟨*S*⟩ (averaged along 10 ns simulations) calculated across the channel heights. The water molecules are classified as close to the capillary wall (bottom and top separately for asymmetric channels) or belong to the middle layers using the water density profiles (Figure S4).

Figure 3a shows that, irrespective of channel chemistries and heights, there is a slight tendency toward water anti-alignment for the water molecules closest to the channel walls. The results can be rationalized by looking at the oxygen and hydrogen density profiles (see Figure S4), which all display a greater density of oxygen atoms in the water layer closest to the channel walls compared to hydrogen atoms. In the two largest channels, the orientation of the water in the middle layers is as expected less influenced by the channel walls leading to an order parameter closer to zero.

The small effect that the chemistry of the wall has on the water structure is confirmed by the calculation of the average number of hydrogen bonds per water molecule, ⟨*N*_HB_⟩, displayed in Figure 3b. Both top and bottom layers for each channel chemistry and height show similar values of ⟨*N*_HB_⟩. These are below the bulk value of 3.6^45^ due to the structural order the water molecules closest to the walls experience, limiting molecule orientation and thus the number of hydrogen bonds each molecule can form. In the 1.68 and 2.37 nm channels, in the layers immediately adjacent to the contact layers, there is a further decrease in the average hydrogen bond number in agreement with our previous work.^58^ In the largest channels (*H* = 2.37 nm), those water molecules in the middle display a value closest to that of bulk water (as it is the case for the density).

### Volumetric Flow Rate

To compare the simulated flow data with the experimental results, the magnitude of the external field, Δ*P* in this case which is the pressure difference across the channel, needs to be carefully chosen to ensure to be within the linear response regime where the velocity of the water molecules in the proximity of the capillary wall, *u*_slip_, and the fluid shear stress at the wall, τ_w_, are linearly related. Both τ_w_ and *u*_slip_ can be calculated directly from the values of Δ*P* and volumetric flow rate, *Q*. The wall shear stress is related to Δ*P* via the force balance
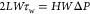
4where *L* is the channel length and *W* is the channel width. Here, the r.h.s. is the force exerted by the fluid in the direction of the flow and the l.h.s. represents the tangential force exerted on the walls of the capillary, resulting in
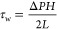
5

As the water/graphite interface slip length, *b*, is of the order of 50 nm^28^ and thus much larger than the capillary height, the flow generated in these nanocapillaries is a “plug flow” where the fluid velocity profile across the capillary is uniform. Although the water/hBN interface is known to have a lower slippage, the resulting slip length has been calculated to be around 7 nm for defect-free hBN,^22^ thus still larger than the largest channel height considered here and therefore a “plug flow” can also be assumed for channels containing hBN. The value of *u*_slip_ can thus be directly calculated from the value of *Q* from
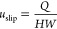
6

**Figure 4:**
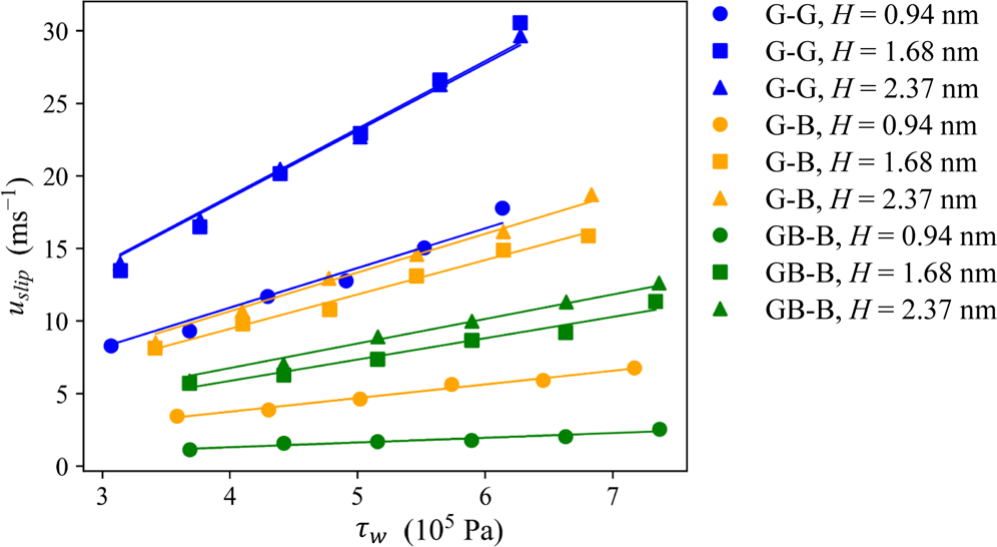
Velocity at the wall (*u*_slip_) vs wall stress (τ_w_) in the linear flow regime for all channel heights and chemistries. For channel labels, see the Methods section. Error estimates obtained by block averages are too small to be viewed on the plot.

Figure S5 in the Supporting Information shows the value of *u*_slip_ plotted against τ_w_ for all capillary widths and surface chemistries. In agreement with recent experiments and with what was observed for nanotubes,^16,32^ capillaries containing hBN have a lower volumetric flow rate. Here, we observe that this reduction is more evident for smaller channels and for those containing hBN on both capillary walls. In the linear regime (Figure 4), the wall stress and slip velocity are linearly related and the proportionality constant is the friction coefficient, λ, τ_w_ = λ·*u*_slip_.

**Figure 5:**
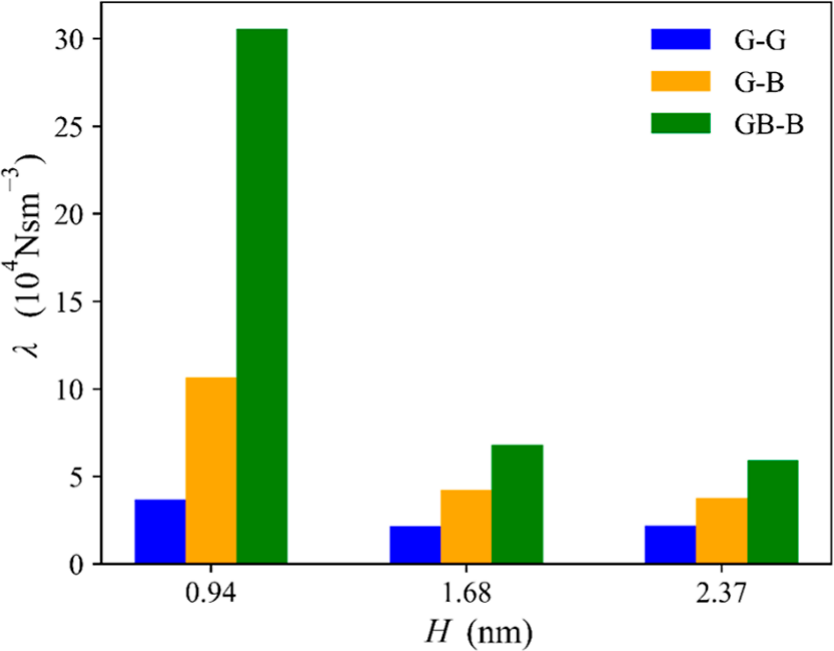
Liquid/solid friction coefficient, λ, obtained from the linear fitting of the data of Figure 4. For channel labels, see the Methods section.

By linear regression of the data presented in Figure 4, we extracted the friction coefficients, whose values are reported in Figure 5 and Table S1 in the Supporting Information. Several observations can be made by looking at the friction data. First, the values of λ calculated for the graphite channels agree well with those previously reported by us and others calculated using either EMD or NEMD simulations^28,59^ but in the latter case with the constrained fitting of the velocity profiles rather than the volumetric flow data as we have done here. The agreement confirms the robustness of the methods to calculate solid/liquid friction coefficients and the reproducibility of the equilibrium and nonequilibrium procedures. Moreover, the simulations show that the value of λ is fairly similar for the two channels with the largest height but becomes significantly larger as the height of the channels is decreased. This is in good agreement with our previous work on graphitic channels,^28^ where the data show that λ is not only a parameter dependent on the chemical nature of the surface and liquid but is also affected by the degree of confinement of the channel. The results also show that the inclusion of hBN layers in the capillary walls substantially increases the friction coefficients, but such an increase is much larger in the channels with the smallest heights with hBN on both walls and decreases with increasing *H*. The friction coefficients calculated for the *H* = 0.94 nm G–B (λ_GB_) and GB–B (λ_GBB_) channels are, respectively, approximately three and ten times the G–G channel (λ_GG_). For larger channels, the increase is, however, less pronounced, and it is the lowest in the largest capillary where λ_GB_(λ_GBB_) ≈ 1.7(2.7)λ_GG_. The extent of this increase is in line with previous results obtained for bulk liquid,^33,34^ although it is worth noting that the values calculated here using the volumetric flow data are an average value between two interfaces, water/graphite and water/hBN or water/hBN-graphite.

**Figure 6:**
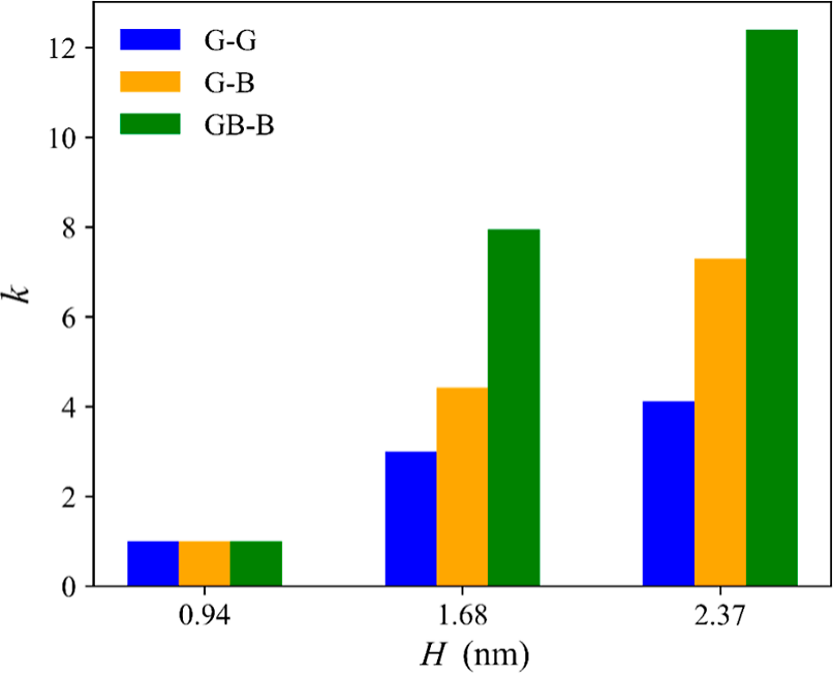
Normalized (with respect to the value calculated in the smallest channel of the corresponding chemistry) permeability, *k*, as a function of channel height, *H*, and channel chemistry. For channel labels, see the Methods section.

The substantial reduction of λ_GBB_ as *H* increases leads to a larger increase in the volumetric flow rate in larger channels containing hBN compared to the one observed in pure graphitic ones and for a similar driving pressure. Figure 6 shows how much *Q* increases as *H* increases for each channel chemistry, comparing the values of the intrinsic permeability, *k*, calculated for the highest value of Δ*P* in the linear regime, normalized by *k* calculated for the smallest channel (*H* = 0.94 nm). The calculation is done separately for each chemistry channel. The permeability can be calculated from the Darcy law as
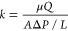
7where μ is the water bulk viscosity and *A* = *HW* is the cross-sectional area of the channel. As we plot the normalized values of *k*, the actual value of the viscosity is not relevant, assuming that the viscosity does not change with the degree of confinement. The plot shows that as the channel height increases to *H* = 2.37 nm, the water permeation increases four times in the graphitic capillary (G–G) but by more than 12 times in the mixed channel (GB–B). These results show that although for capillaries of the same size the volumetric flow rate is larger in graphitic channels than in any others containing hBN (Figure 4), *Q* increases much quicker with the channel height in the latter than in the former.

**Figure 7:**
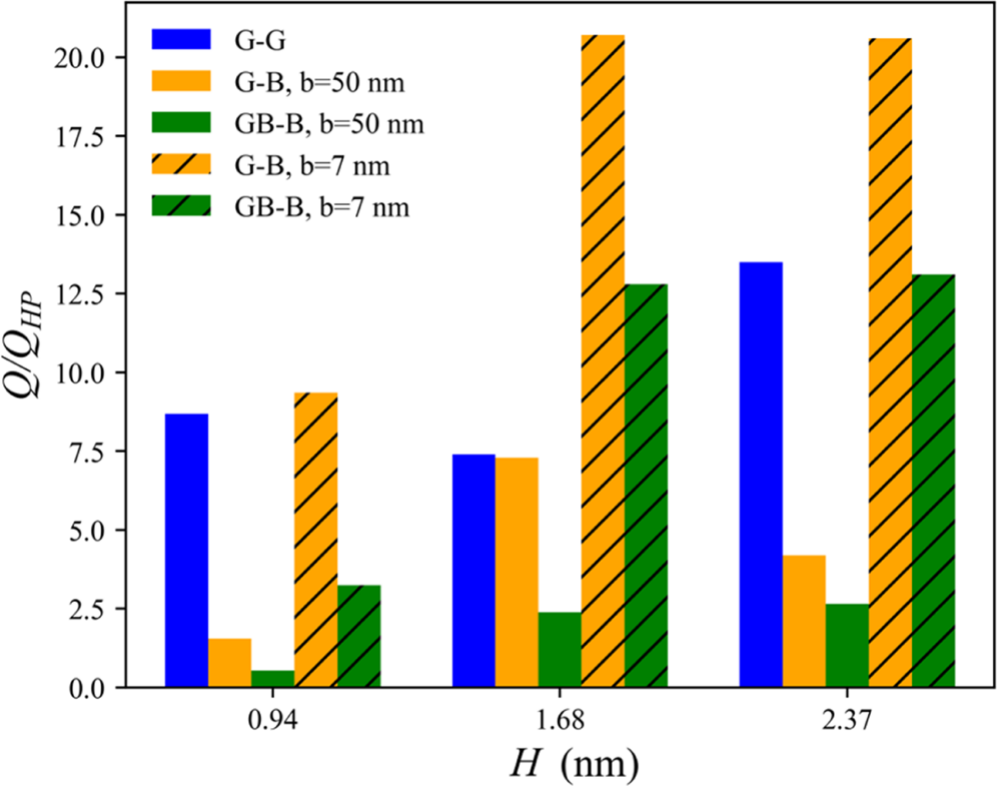
Comparison of the ratio of the volumetric flow rate calculated using eq 8 (*Q*_HP_) and as obtained from MD simulations (*Q*) for each channel chemistry at heights. For channel labels, see the Methods section.

Finally, we compare the values of *Q* calculated from the MD simulations (*Q*) with those predicted by the Hagen–Poiseuille law (*Q*_HP_) for unidirectional flow in a narrow channel subject to wall slip
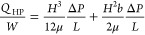
8where *b* is the slip length and μ is the water viscosity (see the Supporting Information about the derivation of equation 8). Figure 7 reports the comparison between *Q* calculated from the MD simulations and *Q*_HP_ predicted using eq 8 using the viscosity of the TIP4P/2005 water model (0.855 mPa s) and *b* = 50 or 7 nm depending on the channel chemistry. For the mixed chemistry channels (G–B and GB–B), the calculations are done using both values of *b*, for comparison. The results indicate that for nanocapillaries, the Hagen–Poiseuille theory underestimates the flow rate calculated from the simulations and the underestimation is greater the larger the channel. In the mixed channels, the theory underestimates the *Q* values even if we assume the largest slip length of 50 nm.

## Summary and Conclusions

In this work, we have shown that the mass (diffusion) and momentum (flow) transfer in nanoconfined water are decoupled and not directly relatable with the wettability of the bulk materials making up the pores’ walls. By means of modeling the mass diffusion and volumetric flow rate of water confined in 2D capillaries of different sizes containing graphene and hBN, we found that water freely diffuses in all capillaries with the same diffusion coefficient irrespective of the hBN content for channels larger than 0.94 nm. The effect of the capillary chemistry wall is shown only in the smallest channels, where the water diffusion is affected by the interactions with both walls and the presence of hBN reduces the water diffusion. The calculation of the diffusion coefficient of the only interfacial water indicates that the diffusion is homogeneous across the capillary and that the dynamics of the interfacial molecules is only very mildly affected compared with that of molecules in the middle of the channel. While the presence of hBN sheets in the wall capillary does not affect the water diffusion for a capillary height larger than 0.94 nm, it greatly reduces its volumetric flow. In agreement with previous simulations and experimental data, we found that the presence of hBN reduces the flow due to the increase in the liquid/solid friction coefficients. Such an increase is, however, dependent on the degree of confinement, and therefore, the water flow is affected differently depending on the size of the capillary. We observe that the water permeation increases three times faster in larger channels containing hBN than those containing only graphite. The simulations also show that the flow rate in these nanochannels is larger (up to 20 times) than that predicted by the Hagen–Poiseuille theory for a plug flow, even assuming a large slip length. The mismatch with the theory is likely due to the confinement-dependent value of the friction coefficient and possibly water apparent viscosity. While a direct comparison between the simulated flow data and the experimental ones (when available) might not be quantitative due to possible vacancies in the hBN sheet, quantum effects in the graphite/water friction coefficient, or surface contamination that affects its wettability, our conclusions regarding the relationship between the water self-diffusion coefficient, its flow rate, and the wall capillary wettability in confinement are general and should be valid for different chemical systems. In particular, due to the dependence of the hBN/water friction coefficient on the lattice defects,^37^ a systematic study on how the density of such defects could affect the flow and diffusivity data would be interesting for real applications. Finally, in this work, the density of the water within the channel was obtained assuming that the slits were in equilibrium with a liquid water reservoir; however, similar studies could be carried out varying the water channel density taking into account that these nanopores could be instead in contact with environments with different partial pressure.^60^

## Supporting Information Available

Simulations to decide the channel heights, plots of the density profiles across the channels, plots of the slip velocities as a function of wall stress to identify linear regime, plot of the flow rate with and without thermostat applied, value of the friction coefficients, and analytical derivation of equation 8 (PDF)
