# Supplementary material for: Relationship between Capillary Wettability, Mass, and Momentum Transfer in Nanoconfined Water: The Case of Water in Nanoslits of Graphite and Hexagonal Boron Nitride
Source: ACS Appl Mater Interfaces. 2024 Oct 8;16(41):56316–24. doi: 10.1021/acsami.4c10738 (PMC11492258; doi:10.1021/acsami.4c10738)
Supplement: Supplementary file 1 — am4c10738_si_001.pdf [file am4c10738_si_001.pdf]

Supporting Information

Relationship between capillary wettability, mass and momentum transfer in nano-confined water: the case of water in nanoslits of graphite and hexagonal boron nitride

Lois Smith,[a,e] Zixuan Wei,[b] Christopher D. Williams,[c] Mara Chiricotto,[d] Claudio Pereira da Fonte,[e] Paola Carbone[*a]*

[a] *Department of Chemistry, University of Manchester, Oxford Road M13 9PL, Manchester (UK)*

[b] *Department of Chemistry, University of Liverpool, Crown Street, L69 7ZD, Liverpool (UK)*

[c] *Division of Pharmacy and Optometry, School of Health Sciences, University of Manchester, Oxford Road, M13 9PL, (UK)*

[d] *The Hartree Centre, STFC Daresbury Laboratory, Warrington WA4 4AD, (UK)*

[e] *Department of Chemical Engineering, University of Manchester, Oxford Road M13 9PL, Manchester (UK)*

[*] Corresponding author: paola.carbone@manchester.ac.uk

Channel Height Validation

Since nanocapillaries are most frequently constructed using several graphene spacer layers, and the capillary walls are inherently flexible, not all capillary heights are mechanically stable. In addition, stable capillary heights will depend on the number of sheets in the spacer layer, the width of nanocapillary and the nature of the confined fluid. In our previous study, [1] MD simulations using extended models were employed to establish the stable capillary heights corresponding to different numbers of spacer sheets in a graphene assembly. To establish whether changing the composition of the base (e.g. from graphene to hBN) substantially effects the equilibrium capillary height, additional MD simulations using extended nanocapillary models were performed.

Briefly, each nanocapillary assembly consisted of a base of 10 larger sheets stacked in the *z*-direction with *x*- and *y*-dimensions of 150 nm and 6 nm, respectively. Two smaller "spacer" graphene sheets with *x*- and *y*-dimensions of 20 nm and 6 nm were stacked on top of the base slab. A second slab of 10 flexible graphene sheets was stacked on top of the spacer sheets. Finally, the capillary was filled with 17862 water molecules to achieve a 2D water density of 21.5 molecules nm^{-2}. The entire assembly was placed at the bottom of a simulation cell with length in the *z*-direction of 12 nm to ensure the base and top slabs could not interact through the periodic boundary.

This set up (Figure S1) is intended to replicate the exact dimensions of an experimentally fabricated device and creates a narrow nanocapillary, periodic in one dimension and with a width of 130 nm. Four different base slab compositions were investigated: i) 10 rigid graphene sheets, ii) 10 flexible graphene sheets, iii) 10 rigid hBN sheets and iv) 9 rigid graphene sheets plus one rigid hBN sheet.

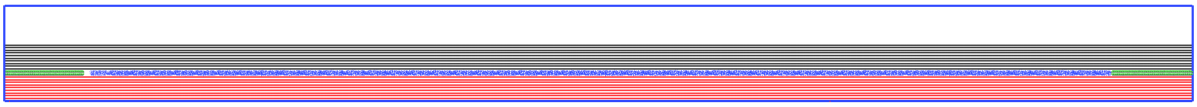

Figure S1 Extended nanocapillary models employed in this study. The assembly is comprised of a base slab containing 10 sheets (red), two graphene spacers (green), top slab containing 10 graphene sheets (black) and water inside in the nanocapillary (blue).

An extended short-range cut-off of 1.7 nm, switched smoothly to zero at 2.0 nm, was employed. The extended cut-off ensures interactions between the base and top slabs are included. This is because these interactions are not negligible when summed over the entire sheet surface area at the distances normally employed for cut-off (~1.2 nm) in MD simulations. The CHARMM potential was used to model all bonded interactions in the flexible graphene sheets. All other simulation parameters were as described in the main text. Each system was simulated for 5.1 ns and the capillary height probably distributions, $P(H)$ were calculated over the final 5 ns.

Due to the flexibility of the graphene sheets, capillary walls are permitted to relax and the nanocapillary height to adopt a different value from the initial state of 1.0 nm. The simulations confirm that, when 2 graphene spacers are employed, the initial capillary height of 1.02 nm is not stable and the walls sag, consistent with our previous study [1] (Figure S2). Although the heights vary between 0.9 and 1.0 nm, the most probably equilibrium nanocapillary height was found to be 0.94 – 0.95 nm, corresponding to a confined water bilayer, irrespective of the chemistry or rigidity of the base slab.

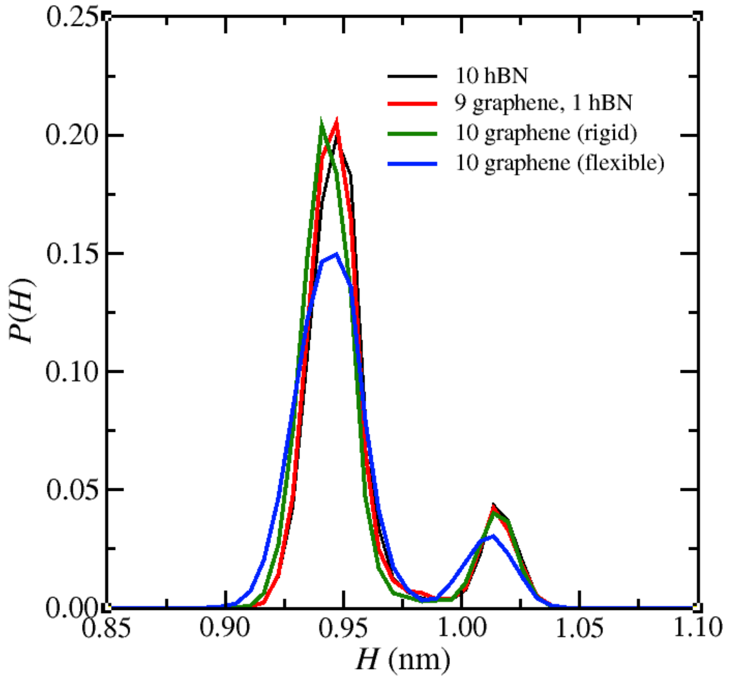

Figure S2 Capillary height probably distribution, $P(H)$ calculated using distances, in the z-direction, between nearest neighbour carbons, in the x, y-plane of the top sheet in the base slab and the bottom sheet of the top slab. The colours represent different base slab compositions and the feature at ~1.1 nm is associated with the spacer region.

NVE simulations

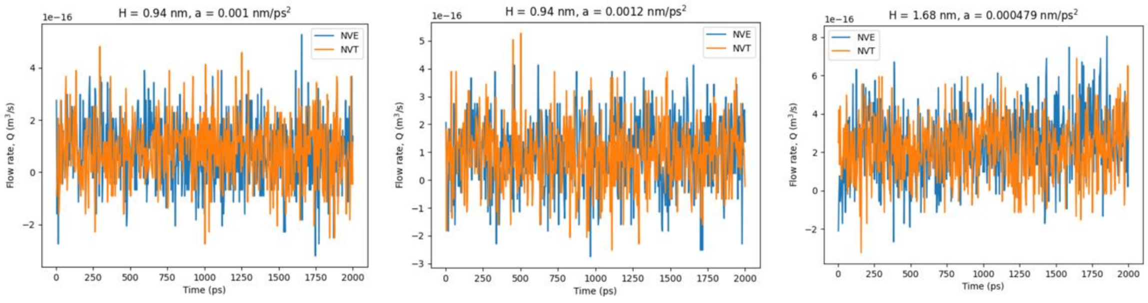

Figure S3. Comparison of the flow rate, Q, obtained from simulations of the graphitic channel systems with (NVT ensemble) and without (NVE ensemble) the thermostat for three acceleration values, a, all within the linear regime, and two channel heights, H.

Channel Densities

The channel oxygen (OW) and hydrogen (HW) number density profiles normalised by species number, N_{sp}, for each channel height and chemistry are displayed in Figure S3. The positions of the OW peaks are used to determine the water molecules present in each water layer.

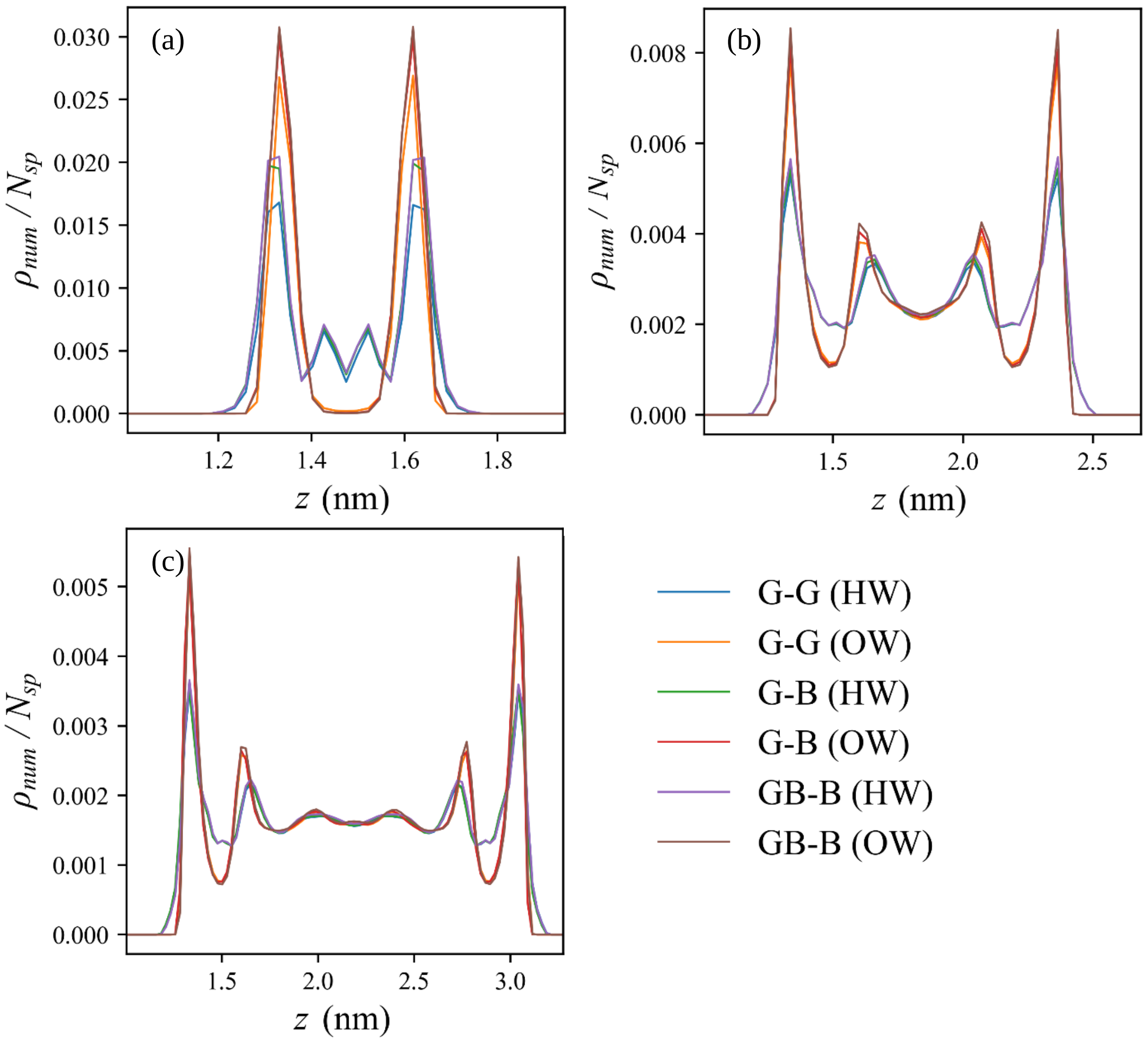

Figure S4. Oxygen (OW) and Hydrogen (HW) number density, ρ_{num}, profiles across the channels normalized by the number of species (OW or HW) in the capillary, N_{sp}, for (a) H=0.94 nm, (b) H=1.68 nm and (c) H=2.37 nm.

Linear Flow Regime

Since the wall stress, τ_w, is linearly dependent on the channel height, H, and the pressure difference applied across each channel, ΔP, (see Equation 5 in the main text) the range of accelerations applied to water molecules had to be carefully considered in order to ensure values of τ_w remained low enough to be in the linear flow regime. Figure S4 displays channel slip velocity, u_{slip}, against τ_{wl} for all combinations of channel height and chemistry. The accelerations applied across each channel were in the range of 1×10^{12} ms^{-2} to 2×10^{12} ms^{-2}. This produces values of u_{slip} which are linearly proportional to τ_w and, hence, fall within the linear flow regime for the 0.94 nm channel only. The 1.68 nm and 2.37 nm channels deviate from this behaviour, with u_{slip} and τ_w displaying a power 2 polynomial relationship and, as such, the pressure difference across these channels must be reduced to achieve linear flow. This behavior is consistent across all channel chemistries. To compensate, we reduced the range of accelerations applied to the 1.68 nm and 2.37 nm channels by approximately an order of magnitude compared to those applied to the 0.94 nm channels. Through this, we achieved similar values of wall stress across all simulations which fall well within the linear flow regime (see Figure 4 of the main text).

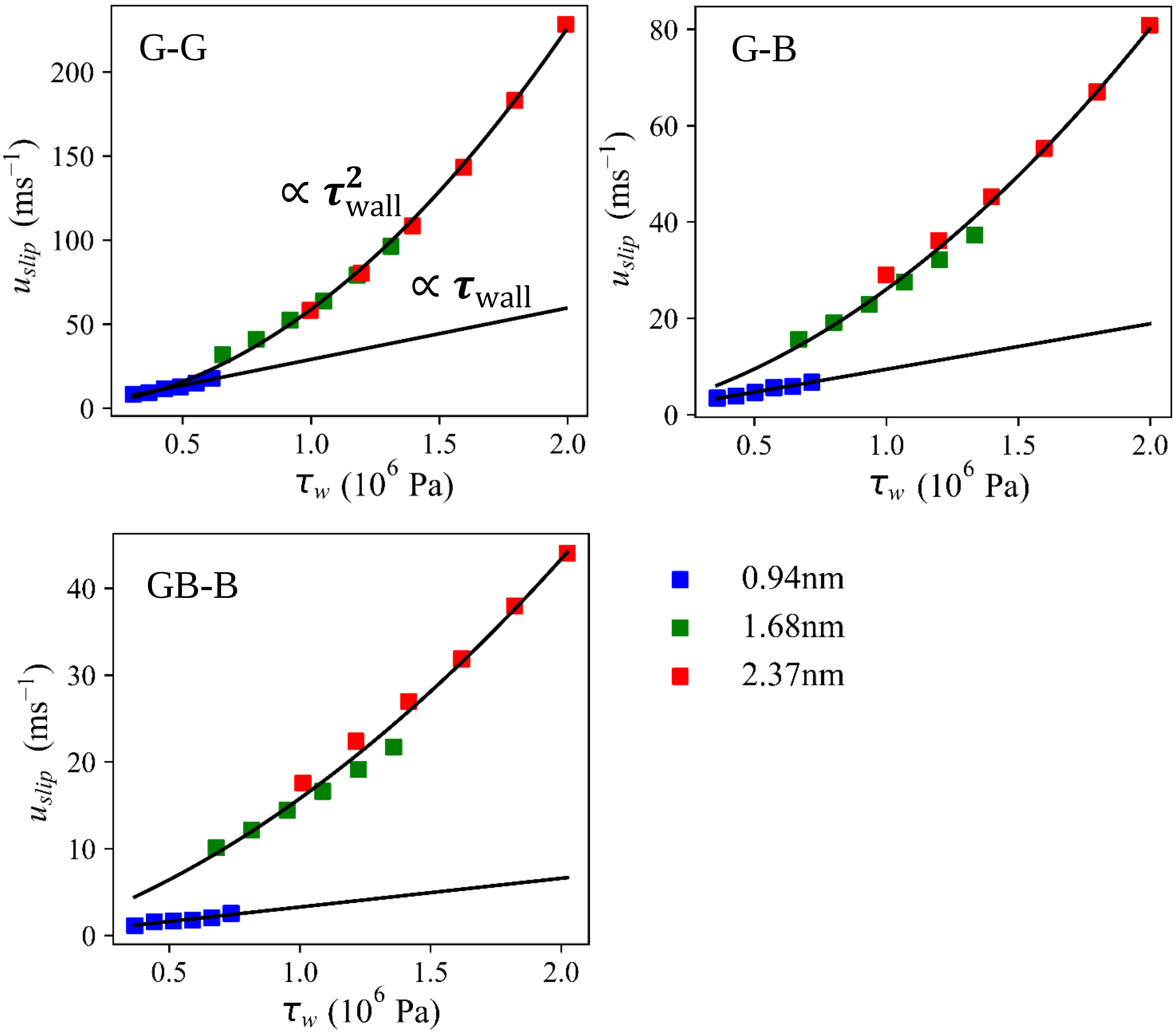

Figure S5 Plots of channel slip velocity against tangential wall stress for each channel chemistry. Values are displayed for channels of heights, H=0.94 nm (blue), H=1.68 nm (green) and H=2.37 nm (red). Linear and quadratic polynomial fits to the data are displayed in black. Error estimates determined by block averages are too small to be displayed on the plot.

Friction Coefficients

Table S1. Liquid/solid friction coefficient, λ, obtained from the linear fitting of data in Figure 4 of the main text. R-squared values for the linear regression fit are of the order ~10 Nsm^{-3}.

H **[nm]**	**Capillary type**	λ **(×10^4 Nsm^{-3})**
0.94	G-G	3.65
1.68		2.15
2.37		2.16
0.94	G-B	10.6
1.68		4.22
2.37		3.75
0.94	GB-B	30.56
1.68		6.79
2.37		5.91

Derivation of equation 8

Assuming that the liquid behaves as a continuum between two parallel walls separated by a distance H, its flow can be described using the momentum balance equation

$$\mu \frac{\mathrm{d}^2 u}{\mathrm{d}y^2} = \frac{\Delta P}{L}$$

where μ is the viscosity of the fluid, u is the velocity in the direction parallel to the two walls, $y \in \left[-\frac{H}{2}, \frac{H}{2}\right]$ is the Cartesian coordinate normal to the walls and $\Delta P/L$ is the uniform pressure gradient in the channel of length L. The equation is subject to the following boundary conditions

$$\begin{cases} \frac{\mathrm{d}u}{\mathrm{d}y} = 0 \text{ at } y = 0 \text{ (Symmetry assumption)} \\ u = b\frac{\mathrm{d}u}{\mathrm{d}y} \text{ at } y = \frac{H}{2} \text{ (Slip condition)} \end{cases}$$

where b is the slip length at the solid boundary. By integration and application of the boundary conditions, we obtain the particular solution

$$u(y) = \frac{\Delta P}{2\mu L}\left[\left(\frac{H}{2}\right)^2 - y^2 + bH\right].$$

The flow rate, Q, per unit width, W, of the channel can then be obtained by integrating the velocity profile in the channel

$$\frac{Q}{W} = 2\int_0^{\frac{H}{2}} u(y)\,\mathrm{d}y = \frac{\Delta P\, H^3}{12\mu L}\left[1 + \frac{6b}{H}\right].$$

References

(1) Williams, C. D.; Wei, Z.; Shaharudin, M. R. B.; Carbone, P. A molecular simulation study into the stability of hydrated graphene nanochannels used in nanofluidics devices. *Nanoscale* **2022**, *14* (9), 3467-3479.
